# Associations of single and multiple vitamin exposure with childhood eczema: data from the national health and nutrition examination survey

**DOI:** 10.3389/fped.2024.1328592

**Published:** 2024-05-15

**Authors:** Fang Wang, Xiaolie Wang, Jiayan Wang, Biqing Liu

**Affiliations:** ^1^ Department of Dermatology, Naval Hospital of Eastern Theater of PLA, Zhoushan, Zhejiang, China; ^2^ Department of Emergency, Naval Hospital of Eastern Theater of PLA, Zhoushan, Zhejiang, China

**Keywords:** eczema, serum vitamins, co-exposure, Bayesian kernel machine regression, quantile g-computation

## Abstract

**Aim:**

The association between vitamins and eczema has garnered attention, yet few studies have evaluated the effects of co-exposure to multiple vitamins on this condition. This study aims to assess the association of vitamin mixtures with eczema in children.

**Methods:**

This cross-sectional study analyzed data from 2,244 children aged 6–17 years from the National Health and Nutrition Examination Surveys. Eczema served as the primary outcome. Six serum vitamins, namely, vitamins A, B_6_, B_12_, C, D, and E, were the main variables. Weighted multivariate logistic regression was adopted to analyze the association between each serum vitamin and eczema. Odds ratios (OR) with a 95% confidence interval (CI) were calculated. Bayesian kernel machine regression (BKMR) analysis and the quantile g-computation (qgcomp) model were used to evaluate the association of co-exposure to multiple vitamins with eczema.

**Results:**

In total, 10.83% of children (*n* = 243) developed eczema. After adjusting for confounding factors, we observed that compared with the reference group (vitamin B_12_ with second quartile), the OR for eczema was 0.604 (95% CI: 0.373–0.978, *P* = 0.041) for the first quartile of vitamin B_12_. Both BKMR analysis and the qgcomp model consistently showed that co-exposure to the six vitamins was positively correlated with the risk of eczema, with vitamin B_6_ contributing most to the overall effect. In BKMR analyses, we observed an interaction between vitamins B_6_ and B_12_ concerning eczema risk.

**Conclusion:**

Co-exposure to vitamins A, C, B_6_, B_12_, D, and E was found to be associated with an increased risk of eczema in children, with vitamin B_6_ as the greatest positive contributor driving the overall effect.

## Introduction

Eczema, also known as atopic eczema/atopic dermatitis, is a prevalent skin disorder in children characterized by impairment of the skin barrier and pruritus (1). Its prevalence in children has been reported to range from 10.6% to 35.7% (2). Eczema can be accompanied by pruritus, sleep disturbances, and numerous comorbidities, which significantly affect the quality of life of children and impose a substantial financial burden on families (3). Therefore, understanding the factors associated with eczema is crucial for preventing its occurrence.

Vitamins are essential organic compounds necessary for maintaining normal physiological functions in humans and animals and play a crucial role in processes such as growth, metabolism, and development (4). Research suggests that vitamins may be involved in the pathogenesis of eczema due to their effects on the epidermis or the immune system (5). A study highlighted the prevalence of micronutrient deficiencies in children with atopic dermatitis (6). Furthermore, the results of animal studies have demonstrated that vitamin A deficiency may exacerbate atopic dermatitis by augmenting Th2-mediated inflammation and activating mast cells (7). In the study of Xiang et al. (8), it was discovered that both vitamin A and vitamin D levels were negatively related to SCORing Atopic Dermatitis (SCORAD) scores and that vitamin A and vitamin D co-deficiency might exacerbate the severity of atopic dermatitis in children. However, not all vitamins confer benefits on immunity and eczema management. A birth cohort study revealed an association between elevated levels of circulating vitamin B_12_ in pregnant women and an increased risk of atopic dermatitis in their offspring (9). In addition, high levels of circulating vitamin C, recognized as an antioxidant nutrient, may have a pro-oxidation effect (10). While numerous studies have investigated the correlation between vitamin levels and the onset and progression of eczema, existing research has predominantly focused on evaluating the impact of individual vitamins, neglecting to consider the potential effects of concurrent exposure to multiple vitamins. In fact, people are commonly exposed to combinations of vitamins, which may exhibit synergistic or antagonistic interactions (11, 12).

In this study, we utilized the data from the National Health and Nutrition Examination Survey (NHANES) to assess the individual and mixed associations of the six vitamins (A, C, B_6_, B_12_, D, and E) with eczema in children, providing certain basis for the prevention and control of childhood eczema.

## Methods

### Study design and population

The NHANES, conducted by the National Center for Health Statistics of the Centers for Disease Control and Prevention, is a cross-sectional sampling survey aimed at evaluating the health and nutritional status of both adults and children in the United States. This survey collected participants' information, including demographic and socioeconomic characteristics, health-related behaviors, health conditions, physical measurements, and laboratory tests administered by trained medical personnel (13). Ethical approval from the Institutional Review Board of the Naval Hospital of Eastern Theater of PLA was waived, because the data were accessed from NHANES (a publicly available database). Similarly, the need for written informed consent was waived due to the retrospective nature of the study.

**Figure 1:**
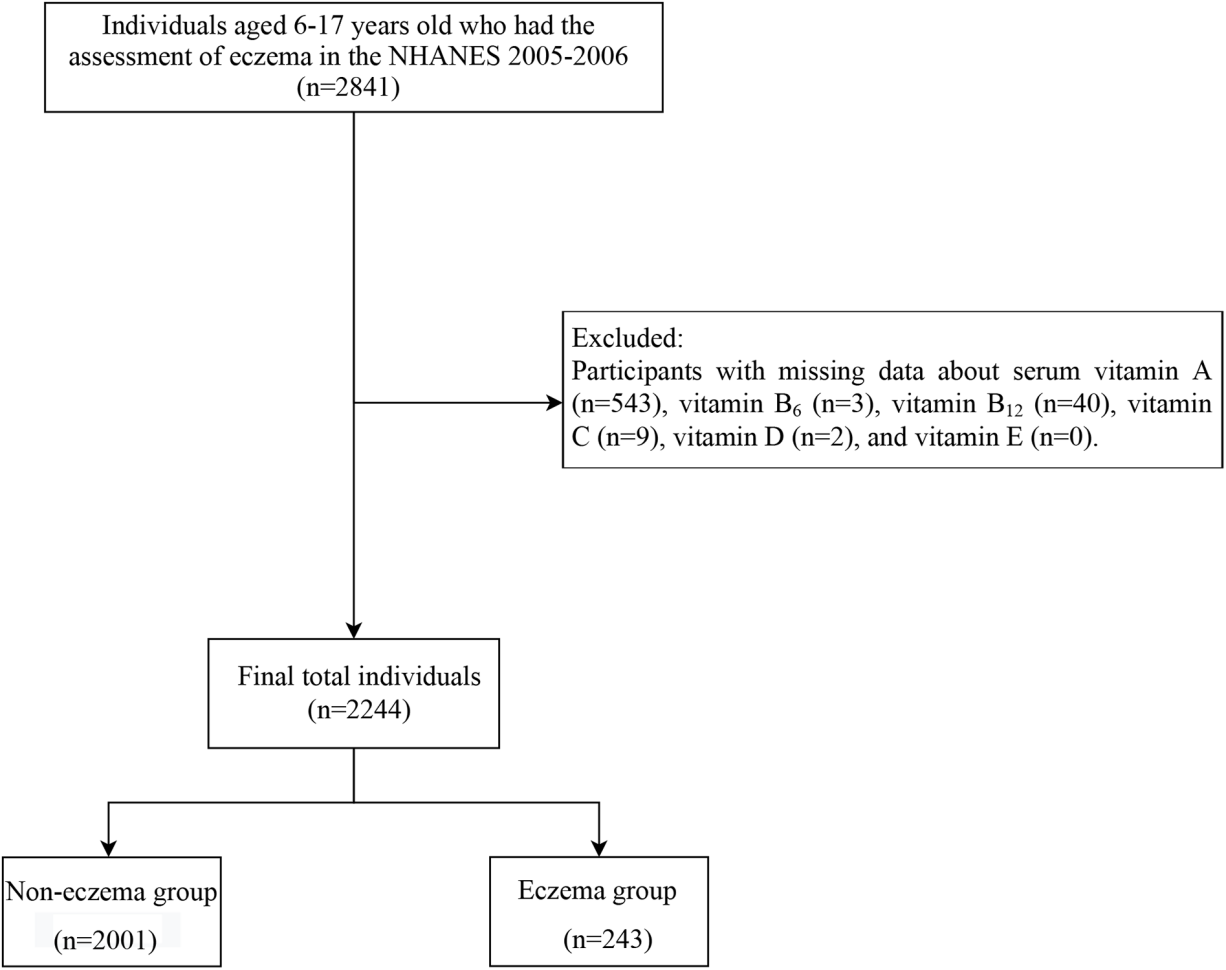
Flowchart for the selection of participants.

In this cross-sectional study, we utilized data from the NHANES from 2005 to 2006. A total of 2,841 individuals aged between 6 and 17 years old who underwent eczema assessment were initially included. We excluded participants with missing data about serum vitamins A, B_6_, B_12_, C, D, and E (*n* = 597). Finally, 2,244 participants remained for subsequent analysis (Figure 1).

### Laboratory measurements

The levels of the six serum vitamins (vitamins A, B_6_, B_12_, C, D, and E) of each participant were assessed through laboratory tests. Serum specimens from each participant were processed, frozen at a temperature of −20°C, and subsequently dispatched to environmental health laboratories for testing purposes. Serum vitamin A, C, D, and E levels were measured using high-performance liquid chromatography (HPLC). Serum vitamin B_6_ and B_12_ levels were measured by using the Bio-Rad Laboratories Quantaphase II radioimmunoassay. The NHANES website provides comprehensive descriptions of all the detection protocols (https://wwwn.cdc.gov/Nchs/Nhanes/2005-2006/VITAEC_D.htm).

Using the three quantiles, each vitamin was divided into three levels in this study: vitamin A (Quantile 1, <1.28 umol/L; Quantile 2, 1.28–1.56 umol/L; Quantile 3, ≥1.56 umol/L), vitamin B_6_ (Quantile 1, <13.80 nmol/L; Quantile 2, 13.80–22.40 nmol/L; Quantile 3, ≥22.40 nmol/L), vitamin B_12_ (Quantile 1, <385.97 pmol/L; Quantile 2, 385.97–551.29 pmol/L; Quantile 3, ≥551.29 pmol/L), vitamin C (Quantile 1, <56.80 umol/L; Quantile 2, 56.80–76.10 umol/L; Quantile 3, ≥76.10 umol/L), vitamin D (Quantile 1, <49.50 nmol/L; Quantile 2, 49.50–64.10 nmol/L; Quantile 3, ≥64.10 nmol/L), and vitamin E (Quantile 1, <16.18 umol/L; Quantile 2, 16.18–19.20 umol/L; Quantile 3, ≥19.20 umol/L).

### Assessment of eczema

Eczema served as the primary outcome of our study. The diagnosis of eczema was determined based on self-reported physician diagnoses, specifically by asking participants, “Have you ever been diagnosed with eczema by a doctor or other healthcare professional?” Those who answered affirmatively were classified as having eczema, whereas those who answered negatively were considered non-eczema individuals (1).

### Collection of possible covariates

The possible covariates included age (years), gender (male and female), race (non-Hispanic Black, non-Hispanic White, Mexican American, and others), poverty-to-income ratio (PIR) (<1 and ≥1), weight (kg), height (cm), body mass index (BMI) (kg/m^2^), low birth weight (yes, no, and unknown), ideal physical activity (yes and no), sedentary time (<3 h, 3–6 h, and ≥6 h), smoking exposure (yes and no), maternal smoking (yes, no, and unknown), hay fever (yes and no), asthma (yes and no), dermatologics (yes and no), C-reactive protein (CRP), and immunoglobulin (IgE, kU/L) (low and high level). The assessment of hay fever information was conducted using the following questions: “Has a doctor or other health professional ever told you that you have hay fever?” or “Have you had an episode of hay fever in the past 12 months?” (14). Asthma diagnosis was determined through the following question: “Has a doctor or other health professional ever told you that you have asthma?” (14). A total IgE level of ≥100 kU/L was defined as a high level (15).

### Statistical analysis

Three sampling weights, namely, WTMEC2YR, SDMVSTRA, and SDMVPSU, were applied. The measurement data were described as mean and standard error (SE), and a weighted two-sample *t*-test was used for comparison between groups. The enumeration data were described as the number and percentages [*n* (%)], and the Rao-Scott chi-square test was used for comparison between groups. *P *< 0.05 was considered statistically significant. SAS 9.4 software (SAS Institute Inc., Cary, NC, USA) and R version 4.2.3 (2023-03-15 ucrt) were used in this study.

A weighted univariate logistic regression model was utilized to explore the confounding factors associated with eczema (Supplementary Table S1). We employed both weighted univariate and multivariate logistic regression models to analyze the association between each serum vitamin and eczema. In this study, odds ratios (OR) with a 95% confidence interval (CI) were calculated. The non-linear association of serum vitamin and eczema (on a continuous scale) was examined by the restricted cubic spline (RCS) regression analysis. To investigate the effects of the six mixed serum vitamins on eczema in children, we employed two mixture analysis methods: Bayesian kernel machine regression (BKMR) and the quantile g-computation (qgcomp) model.

### BKMR analysis

BKMR analysis is a non-parametric Bayesian framework for variable selection, which combines Bayesian and statistical learning methods to iteratively estimate the exposure–response function using Gaussian kernel functions (16). The method enables not only the assessment of the overall mixing effect and the impact of individual components in the mixture but also the evaluation of potential interactions among these components (17). In this study, we used Pearson’s correlation analysis to calculate the correlation coefficient between the two vitamins and construct a correlation coefficient diagram, and the six vitamins were grouped according to the correlation coefficient. Subsequently, the combined effect was determined by comparing mixed vitamins at or above the 60th percentile with the 50th percentile. The group posterior inclusion probability (GroupPIP) and conditional posterior inclusion probability (CondPIP) quantify the likelihood of each group and serum vitamin within each group being included in the model, thereby indicating their respective contributions to the overall effect. BKMR analysis was implemented by using the R package “bkmr.”

### qgcomp model

The qgcomp model is a parameterized linear model that could evaluate the combined effects of individual exposures in different directions (18). The qgcomp noboot function was utilized in this study to assess the overall effects by dividing each vitamin into quantiles, assigning a weighted index (positive or negative) to each vitamin, and thus fitting linear models for eczema. The qgcomp model analysis was conducted by using the R package “qgcomp.”

## Results

### Demographics and characteristics of participants

In total, 2,244 children were included in our study, of whom 1,124 (51.80%) were boys and 1,120 (48.20%) were girls. Table 1 presents the essential demographic and characteristics of all children (*n* = 2,244). The mean (±SE) values of weight and height were 50.20 (±0.84) kg and 151.81 (±0.70) cm for the total population. Among the children, approximately 10.83% (*n* = 243) developed eczema. Children with eczema had lower mean weight and height and were more likely to have hay fever and asthma than those without eczema (Table 1).

**Table 1:** Demographics and characteristics of participants (*n* = 2,244).

Variables	Total (*n* = 2,244)	Non-eczema group (*n* = 2,001)	Eczema group (*n* = 243)	*P*
Vitamin A, μmol/L, mean (± S.E)	1.47 (±0.02)	1.48 (±0.02)	1.40 (±0.02)	0.005
Vitamin B_6_, nmol/L, mean (± S.E)	28.37 (±1.08)	27.75 (±1.22)	32.51 (±1.64)	0.040
Vitamin B_12_, pmol/L, mean (± S.E)	497.09 (±6.19)	490.22 (±6.48)	542.61 (±16.13)	0.008
Vitamin C, μmol/L, mean (± S.E)	67.40 (±1.23)	66.69 (±1.23)	72.11 (±2.50)	0.040
Vitamin D, nmol/L, mean (± S.E)	64.70 (±1.23)	64.35 (±1.17)	67.01 (±2.57)	0.255
Vitamin E, μmol/L, mean (± S.E)	18.72 (±0.17)	18.66 (±0.19)	19.10 (±0.31)	0.234
Age, years, *n* (%)				0.002
<12	836 (45.35)	724 (43.52)	112 (57.41)	
≥12	1,408 (54.65)	1,277 (56.48)	131 (42.59)	
Gender, *n* (%)				0.706
Male	1,124 (51.80)	1,007 (52.08)	117 (49.95)	
Female	1,120 (48.20)	994 (47.92)	126 (50.05)	
Ethnicity, (%)				0.010
Non-Hispanic Black	694 (13.77)	583 (13.18)	111 (17.72)	
Non-Hispanic White	593 (61.86)	511 (61.03)	82 (67.40)	
Mexican American	772 (13.24)	745 (14.70)	27 (3.62)	
Other	185 (11.12)	162 (11.10)	23 (11.27)	
PIR, *n* (%)				0.052
<1	609 (17.71)	560 (18.56)	49 (12.13)	
≥1	1,635 (82.29)	1,441 (81.44)	194 (87.87)	
Weight, kg, mean (± S.E)	50.20 (±0.84)	50.72 (±0.82)	46.77 (±1.54)	0.012
Height, cm, mean (± S.E)	151.81 (±0.70)	152.29 (±0.71)	148.60 (±1.29)	0.011
BMI, kg/m^2^, mean (± S.E)	0.49 (±0.04)	0.49 (±0.04)	0.45 (±0.10)	0.614
Low birth weight, (%)				0.023
No	1,470 (69.86)	1,299 (68.83)	171 (76.66)	
Yes	204 (8.38)	173 (8.03)	31 (10.69)	
Unknown	570 (21.76)	529 (23.14)	41 (12.65)	
Ideal physical activity, *n* (%)				0.033
No	839 (35.98)	730 (34.54)	109 (45.51)	
Yes	1,405 (64.02)	1,271 (65.46)	134 (54.49)	
Sedentary time, *n* (%)				0.794
<3h	1,065 (52.23)	959 (52.17)	106 (52.67)	
3–6h	926 (37.52)	816 (37.37)	110 (38.49)	
≥6h	253 (10.25)	226 (10.46)	27 (8.84)	
Smoking exposure, *n* (%)				0.712
No	1,856 (83.12)	1,662 (83.02)	194 (83.81)	
Yes	388 (16.88)	339 (16.98)	49 (16.19)	
Maternal smoking, *n* (%)				0.040
No	1,460 (64.78)	1,291 (63.57)	169 (72.79)	
Yes	267 (15.37)	233 (15.47)	34 (14.73)	
Unknown	517 (19.85)	477 (20.97)	40 (12.48)	
Hay fever, *n* (%)				0.004
No	2,160 (95.79)	1,940 (96.58)	220 (90.58)	
Yes	84 (4.21)	61 (3.42)	23 (9.42)	
Asthma, *n* (%)				0.001
No	1,876 (82.98)	1,707 (84.55)	169 (72.54)	
Yes	368 (17.02)	294 (15.45)	74 (27.46)	
Dermatologics, *n* (%)				0.123
No	2,215 (98.08)	1,979 (98.41)	236 (95.89)	
Yes	29 (1.92)	22 (1.59)	7 (4.11)	
CRP, mean (±S.E)	0.18 (±0.01)	0.17 (±0.02)	0.23 (±0.10)	0.561
Cotinine, mean (±S.E)	7.56 (±1.08)	8.08 (±1.25)	4.10 (±2.05)	0.134
IgE, *n* (%)				0.142
Low level	1,398 (68.41)	1,265 (69.15)	133 (63.50)	
High level	846 (31.59)	736 (30.85)	110 (36.50)	

PIR, poverty-to-income ratio; BMI, body mass index; CRP, C-reactive protein; IgE, immunoglobulin.

### Effects of individual vitamin on eczema

As shown in Table 2, when considering vitamins as a continuous variable, it was observed that vitamin A level was inversely associated with the odds of eczema after adjustment for all covariates (OR = 0.642, 95% CI: 0.414–0.996, *P *= 0.048). In addition, RCS results indicated that not all vitamins were linearly associated with eczema (Supplementary Figure S1). After conducting weighted univariate logistic regression analysis, we found significant associations between vitamin B_12_ with the first quartile (as a categorical variable) and eczema (Model 1: OR = 0.515, 95% CI: 0.318–0.834, *P *= 0.011) and between vitamin C with the third quartile (as a categorical variable) and eczema (Model 1: OR = 1.650, 95% CI: 1.126–2.419, *P *= 0.014). After adjusting for age, gender, ethnicity, ideal physical activity, hay fever, and asthma, compared with the reference group (vitamin B_12_ with second quartile), the OR for eczema was 0.566 (95% CI: 0.321–1.028, marginal significance) for the first quartile of vitamin B_12._ In the fully adjusted models (Model 3), the OR for eczema was 0.604 (95% CI: 0.373–0.978, *P *= 0.041) for the first quartile of vitamin B_12_. However, we did not find any significant associations between the other five vitamins and eczema among children in the fully adjusted model (*P *> 0.05).

**Table 2:** Association between serum vitamin levels and the risk of eczema.

Variables	Model 1		Model 2		Model 3	
	OR (95% CI)	*P*	OR (95% CI)	*P*	OR (95% CI)	*P*
Vitamin A ^(Ⅰ)^	0.526 (0.341–0.811)	0.007	0.677 (0.378–1.213)	0.153	0.642 (0.414–0.996)	0.048
Vitamin A ^(Ⅰ)^						
Quantile 2	Ref		Ref		Ref	
Quantile 1	0.990 (0.579–1.694)	0.970	0.853 (0.452–1.610)	0.549	0.873 (0.505–1.509)	0.627
Quantile 3	0.628 (0.364–1.085)	0.089	0.707 (0.365–1.370)	0.236	0.686 (0.413–1.140)	0.146
Vitamin B_6_^(Ⅱ)^	1.005 (1.000–1.011)	0.063	1.005 (0.998–1.012)	0.156	1.004 (0.998–1.009)	0.230
Vitamin B_6_^(Ⅱ)^						
Quantile 2	Ref		Ref		Ref	
Quantile 1	0.754 (0.470–1.210)	0.220	0.760 (0.414–1.394)	0.297	0.787 (0.464–1.336)	0.375
Quantile 3	1.213 (0.887–1.659)	0.205	1.163 (0.774–1.745)	0.385	1.111 (0.837–1.476)	0.466
Vitamin B_12_^(Ⅲ)^	1.001 (1.001–1.001)	<0.001	1.001 (1.000–1.001)	0.049	1.001 (1.000–1.001)	0.092
Vitamin B_12_^(Ⅲ)^						
Quantile 2	Ref		Ref		Ref	
Quantile 1	0.515 (0.318–0.834)	0.011	0.566 (0.321–0.996)	0.049	0.603 (0.373–0.974)	0.039
Quantile 3	0.917 (0.624–1.347)	0.635	0.862 (0.546–1.360)	0.455	0.798 (0.566–1.125)	0.198
Vitamin C ^(Ⅳ)^	1.007 (1.000–1.014)	0.038	1.005 (0.997–1.013)	0.175	1.003 (0.997–1.010)	0.300
Vitamin C ^(Ⅳ)^						
Quantile 2	Ref		Ref		Ref	
Quantile 1	1.200 (0.780–1.848)	0.377	1.247 (0.757–2.054)	0.307	1.263 (0.843–1.894)	0.258
Quantile 3	1.650 (1.126–2.419)	0.014	1.498 (0.942–2.382)	0.075	1.361 (0.968–1.914)	0.077
Vitamin D ^(Ⅴ)^	1.007 (0.995–1.020)	0.245	1.005 (0.987–1.024)	0.500	1.005 (0.990–1.020)	0.508
Vitamin D ^(Ⅴ)^						
Quantile 2	Ref		Ref		Ref	
Quantile 1	0.821 (0.500–1.347)	0.405	0.838 (0.451–1.556)	0.496	0.915 (0.528–1.587)	0.752
Quantile 3	1.027 (0.562–1.877)	0.926	0.882 (0.418–1.860)	0.683	0.867 (0.493–1.525)	0.620
Vitamin E ^(Ⅵ)^	1.017 (0.988–1.048)	0.236	1.009 (0.974–1.045)	0.560	1.000 (0.971–1.029)	0.973
Vitamin E ^(Ⅵ)^						
Quantile 2	Ref		Ref		Ref	
Quantile 1	0.839 (0.407–1.729)	0.609	0.863 (0.361–2.065)	0.683	0.872 (0.459–1.658)	0.676
Quantile 3	1.119 (0.646–1.937)	0.666	1.071 (0.579–1.980)	0.786	1.041 (0.640–1.691)	0.872

OR, odds ratio; CI, confidence interval.

Model 1: unadjusted.

Model 2: adjusted for age, gender, ethnicity, ideal physical activity, hay fever, and asthma.

Model 3: For (Ⅰ)—further adjusted for vitamins B_6_, B_12_, C, D, and E based on Model 2.

For (Ⅱ)—further adjusted for vitamins A, B_12_, C, D, and E based on Model 2.

For (Ⅲ)—further adjusted for vitamins A, B_6_, C, D, and E based on Model 2.

For (Ⅳ)—further adjusted for vitamins A, B_6_, B_12_, D, and E based on Model 2.

For (Ⅴ)—further adjusted vitamins A, B_6_, B_12_, C, and E based on Model 2.

For (Ⅵ)—further adjusted vitamins A, B_6_, B_12_, C, and D based on Model 2.

### Effects of mixed vitamins on eczema by BKMR and qgcomp analyses

**Figure 2:**
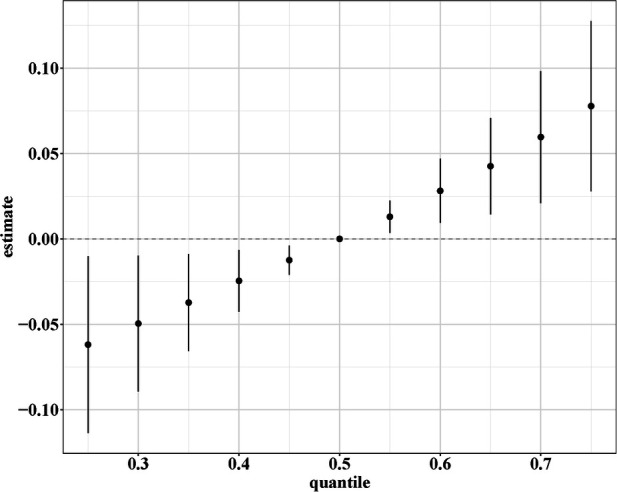
Overall association between the six mixed serum vitamins and eczema in the BKMR analysis. The model was adjusted for age, gender, ethnicity, ideal physical activity, hay fever, and asthma.

BKMR and qgcomp analyses were performed to examine the effects of the six mixed serum vitamins on eczema among children. In the BKMR analysis, we first grouped vitamins A, B_12_, C, and E into Group 1 and vitamins B_6_ and D into Group 2, based on the result of Pearson’s correlation analysis (Supplementary Figure S2). As displayed in Table 3, the GroupPIP of Group 1 was lower than that of Group 2 (0.528 vs. 0.886), and vitamin B_6_ showed the highest contribution to the BKMR analysis (CondPIP = 0.847). Figure 2 indicates the overall association between the six mixed serum vitamins and eczema. Among all participants, the overall exposure–response function indicated a significant and positive association of mixed serum vitamins with eczema. Supplementary Figure S3 presents the univariate estimation of exposure–response functions on the relationship between the vitamin mixtures on eczema. When all other vitamins were at their median levels, positive correlations were found between vitamins B_6_, B_12_, and E with eczema. Additionally, we also found that there may be an interaction between vitamin B_6_ and vitamin B_12_ on eczema risk (Supplementary Figure S4).

**Table 3:** GroupPIP and CondPIP of the six vitamins.

Vitamins	Group	GroupPIP	CondPIP
Vitamin A	1	0.528	0.049
Vitamin B_6_	2	0.886	0.847
Vitamin B_12_	1	0.528	0.492
Vitamin C	1	0.528	0.303
Vitamin D	2	0.886	0.154
Vitamin E	1	0.528	0.155

GroupPIP, group posterior inclusion probability; CondPIP, conditional posterior inclusion probability.

**Figure 3:**
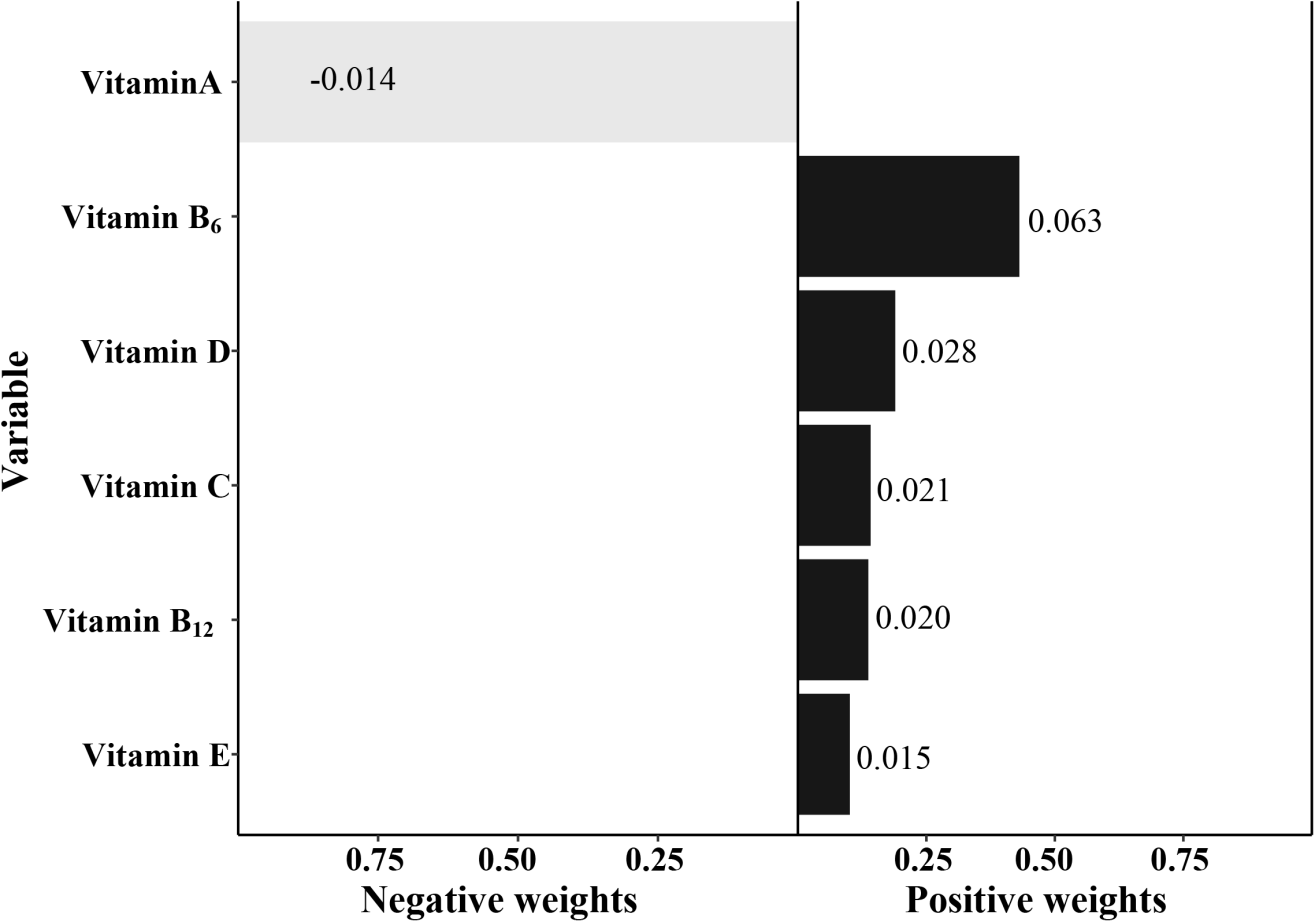
The qgcomp model regression index weights of the six mixed serum vitamins on eczema. The model was adjusted for age, gender, ethnicity, ideal physical activity, hay fever, and asthma.

In the qgcomp model, the mixtures of the six serum vitamins showed a significantly positive association with eczema risk (Table 4, OR = 1.14, 95% CI: 1.01–1.29, *P *= 0.037). Vitamin B_6_ had the greatest positive contribution to the overall associations, followed by vitamin D and vitamin B_12_, respectively (Figure 3).

**Table 4:** The qgcomp model to assess the combined association between the six vitamins and eczema.

Model	OR (95% CI)	*P*
g-computation index	1.14 (1–1.29)	0.037

CI, confidence interval; OR, odds ratio. The model was adjusted for age, gender, ethnicity, ideal physical activity, hay fever, and asthma.

## Discussion

In this study, single and multiple exposures of the six serum vitamins (A, C, B_6_, B_12_, D, and E) on eczema in children were evaluated. The weighted multivariate logistic regression illustrated that a low serum level of vitamin B_12_ was associated with lower odds of eczema in children after adjusting for potential confounders. More importantly, both BKMR analysis and the qgcomp model consistently displayed that mixed exposure to vitamins A, C, B_6_, B_12_, D, and E was significantly positively correlated with eczema in children, with vitamin B_6_ contributing most to the overall effect.

Increasingly convincing evidence displays that vitamins were associated with eczema (5, 19, 20). The pathogenesis of eczema involves immune system dysfunction and impairment of the skin barrier (21). Vitamins are believed to play a crucial role in susceptibility to bacterial and viral infections of the skin (20). Furthermore, vitamins may also impact eczema through their multifaceted effects on altered epidermal barrier function, immune dysregulation, and inadequate bacterial defenses (19). In this study, we found that serum vitamin B_12_ was associated with decreased odds of eczema in children. Vitamin B_12_ is an essential water-soluble micronutrient with antioxidant properties (22). It is reported that topical vitamin B_12_ could be used as a potential treatment option in children with eczema (23). However, in the fully adjusted model, there were no significant associations between the other five vitamins and eczema among children. The potential reason was associated with the origin of the incorporated sample in this study. Further studies are needed to verify the findings.

In previous epidemiological studies, the possible additive and synergistic effects of multivitamin exposure on childhood eczema have been often overlooked (11, 24, 25). However, considering that individuals are typically exposed to multiple vitamins rather than a single one in real-world scenarios, it is imperative to investigate the effects of mixed vitamin exposure on childhood eczema (26). Recently, BKMR analysis and the qgcomp model were widely used to assess the impact of mixed exposure on health (27). Ketema et al. (28) adopted BKMR analysis to show that a mixture of phthalate metabolites was associated with eczema in 7-year-old children. A cross-sectional study found that co-exposure to water-soluble vitamins was associated with a lower risk of metabolic syndrome based on the qgcomp model and that vitamin C provided the greatest negative weight to the qgcomp index (29). In this study, we found a positive association between co-exposure to vitamins A, C, B_6_, B_12_, D, and E and eczema in children, with vitamin B_6_ as the highest positive weighted vitamin. A synergistic effect may be used to explain potential mechanisms of the observed associations between multivitamin exposure and childhood eczema. Vitamins D and A exert a regulatory impact on both the immune system and skin barrier function that were associated with the pathogenesis of eczema (19, 30). Vitamins E and C might influence eczema development through their antioxidant and anti-inflammatory bioactivities (31, 32). The anti-inflammatory and immunoregulatory potential of vitamin B_12_ can lead to dermatological manifestations (33). When multiple vitamins are co-exposed, they can exert their effects simultaneously by utilizing multi-pathways. Further prospective studies are needed to elucidate the specific mechanism. In summary, these findings provide important evidence for exploring the overall effects of serum vitamin mixtures on the risk of eczema.

To the best of our knowledge, the present study is the first to investigate the association of mixtures of the six serum vitamins with eczema risk among children using both BKMR analysis and the qgcomp model, which provides preliminary evidence that co-exposure to high concentrations of vitamins A, C, B_6_, B_12_, D, and E may increase the risk of eczema in children in the United States. Nevertheless, there were some potential limitations to our study. First, we were unable to establish a causal relationship between serum vitamins and eczema due to the cross-sectional design of this study. Second, information on eczema was obtained through self-reported questionnaires, which introduces the possibility of recall bias and may lead to an underestimation of the current prevalence. However, the self-report of eczema has been validated and shows a good correlation with clinical examination results (34). Third, the NHANES does not differentiate between various subtypes of eczema, and the association between vitamins and specific types of eczema may exhibit variations. Lastly, despite trying our best to adjust for the potential confounders, it is important to acknowledge that not all confounding factors could be included (such as a family history of atopic dermatitis and/or other allergic diseases and supplemental vitamin intake). More prospective studies are needed to verify our findings.

## Conclusion

Our findings suggested that a low serum level of vitamin B_12_ may be associated with lower odds of eczema in children. Importantly, co-exposure to vitamins A, C, B_6_, B_12_, D, and E was associated with an increased risk of eczema in children, with vitamin B_6_ as the greatest positive contributor driving the overall effect. However, further prospectively designed studies are warranted to validate our results and explore the underlying mechanisms.

## Data Availability

Publicly available datasets were analyzed in this study. This data can be found here: NHANES database, https://wwwn.cdc.gov/nchs/nhanes/.
