# Supplementary material for: Associations of single and multiple vitamin exposure with childhood eczema: data from the national health and nutrition examination survey
Source: Front Pediatr. 2024 May 15;12:1328592. doi: 10.3389/fped.2024.1328592 (PMC11133564; doi:10.3389/fped.2024.1328592)
Supplement: Supplementary file 1 [file Table1.docx]

**Supplemental Table 1 The Selection of covariates by univariate logistic regression**

| Variables | OR (95% CI) | *P* |
| --- | --- | --- |
| Age |  |  |
| Age<12 | Ref |  |
| Age≥12 | 0.572 (0.418-0.782) | 0.002 |
| Gender |  |  |
| Male | Ref |  |
| Female | 1.089 (0.677-1.752) | 0.706 |
| Ethnicity |  |  |
| Non-Hispanic Black | Ref |  |
| Non-Hispanic White | 0.821 (0.626-1.078) | 0.141 |
| Mexican American | 0.183 (0.098-0.343) | <0.001 |
| Other | 0.755 (0.319-1.788) | 0.491 |
| PIR |  |  |
| <1 | Ref |  |
| ≥1 | 1.650 (0.987-2.758) | 0.055 |
| BMI | 0.967 (0.845-1.108) | 0.605 |
| Low birthweight |  |  |
| No | Ref |  |
| Yes | 1.195 (0.582-2.456) | 0.602 |
| Unknown | 0.491 (0.343-0.703) | 0.001 |
| Ideal physical activity |  |  |
| No | Ref |  |
| Yes | 0.632 (0.414-0.964) | 0.035 |
| Sedentary time |  |  |
| <3h | Ref |  |
| 3~6h | 1.020 (0.675-1.543) | 0.918 |
| ≥6h | 0.837 (0.468-1.499) | 0.522 |
| Smoke exposure |  |  |
| No | Ref |  |
| Yes | 0.945 (0.683-1.306) | 0.712 |
| Maternal smoking |  |  |
| No | Ref |  |
| Yes | 0.832 (0.467-1.480) | 0.502 |
| Unknown | 0.520 (0.369-0.732) | 0.001 |
| Hay fever |  |  |
| No | Ref |  |
| Yes | 2.938 (1.430-6.038) | 0.006 |
| Asthma |  |  |
| No | Ref |  |
| Yes | 2.071 (1.414-3.035) | 0.001 |
| Dermatologics |  |  |
| No | Ref |  |
| Yes | 2.658 (0.701-10.070) | 0.138 |
| CRP | 1.127 (0.781-1.628) | 0.495 |
| Cotinine | 0.996 (0.988-1.004) | 0.300 |
| IgE |  |  |
| Low-level | Ref |  |
| High-level | 1.289 (0.907-1.832) | 0.144 |
| PIR=poverty-to-income ratio; BMI=body mass index; CRP=C-reactive protein; IgE= immunoglobulin. | | |
