# Supplementary figures and images for: Associations of single and multiple vitamin exposure with childhood eczema: data from the national health and nutrition examination survey
Source: Front Pediatr. 2024 May 15;12:1328592. doi: 10.3389/fped.2024.1328592 (PMC11133564; doi:10.3389/fped.2024.1328592)

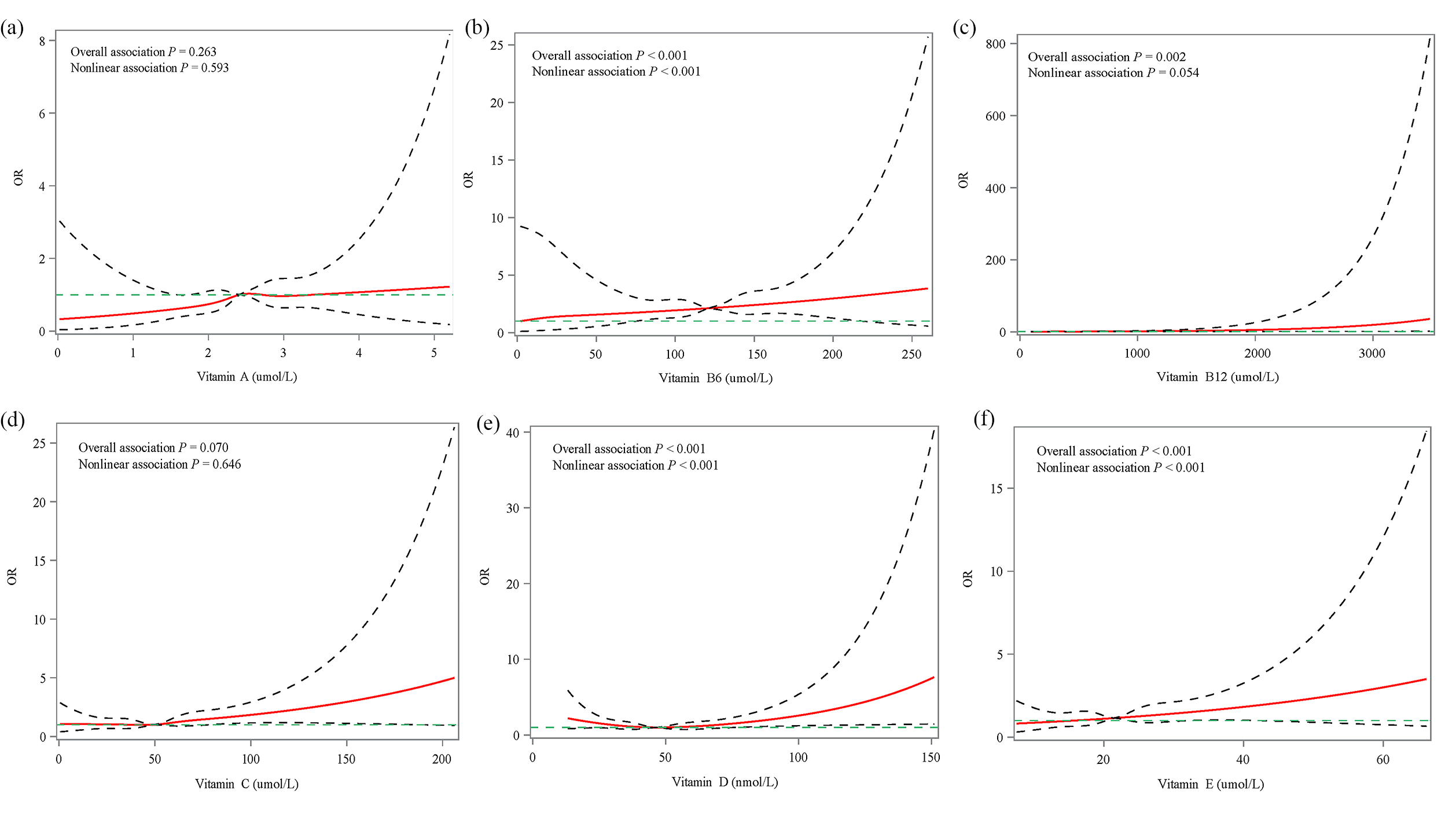

Supplement: Supplementary file 2 [file Image1.tif]

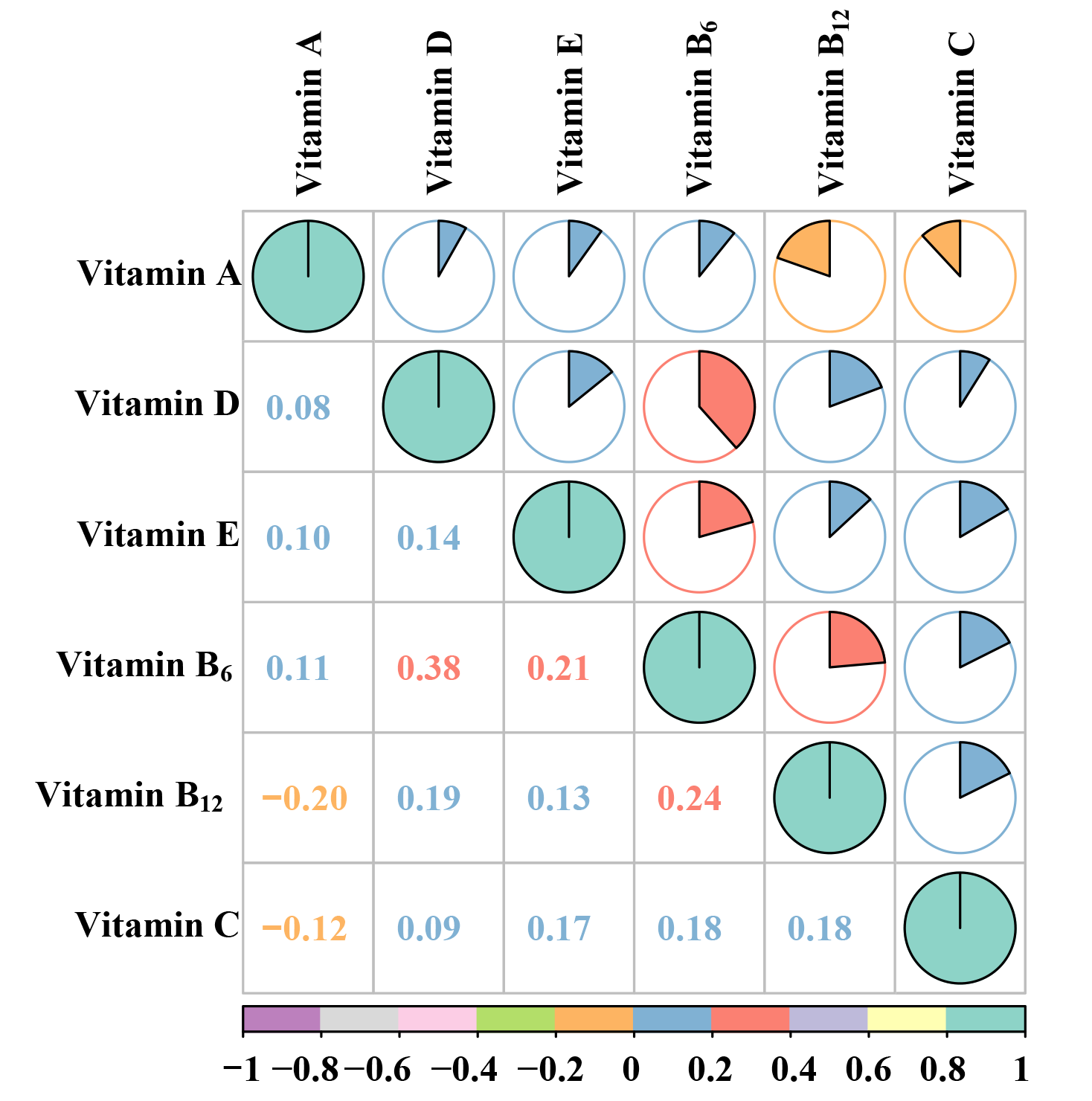

Supplement: Supplementary file 3 [file Image2.tif]

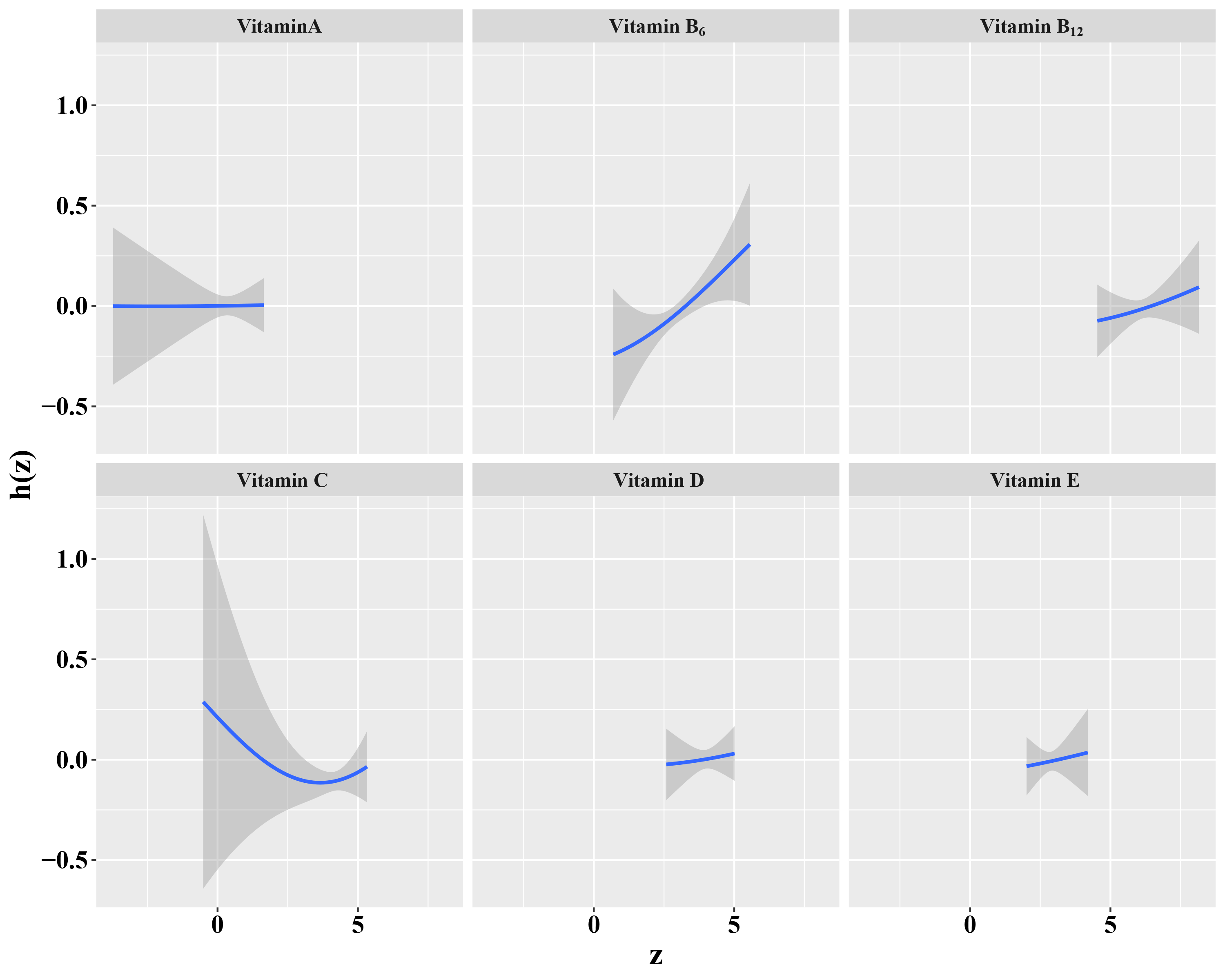

Supplement: Supplementary file 4 [file Image3.tif]

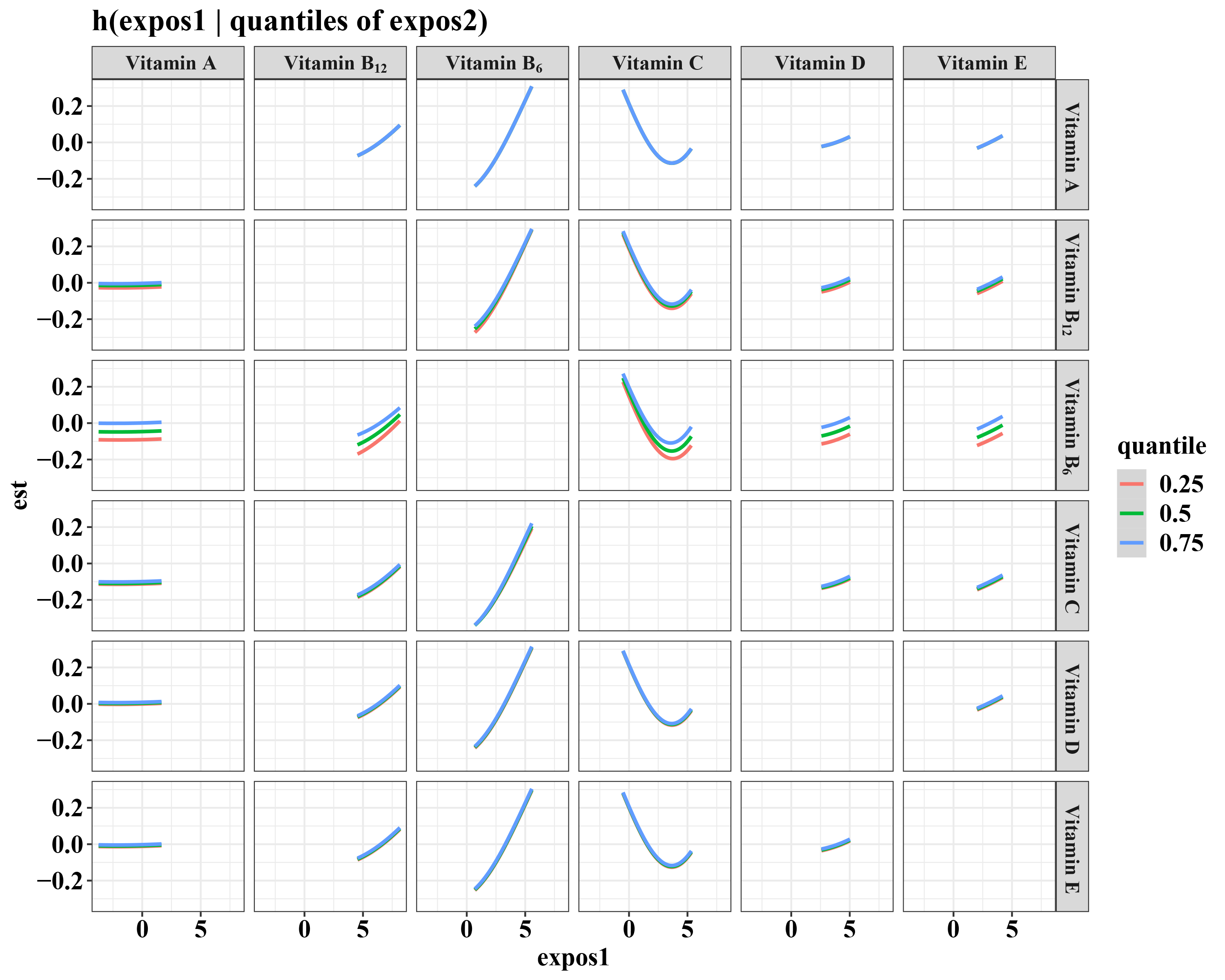

Supplement: Supplementary file 5 [file Image4.tif]
